# The Asylums Board and Sick Children: Inhumanity or Progress?

**Published:** 1913-09-13

**Authors:** C. Thackray Parsons

**Affiliations:** Medical Superintendent Fulham Infirmary.


### THE EDITOR'S LETTER-BOX.

### The Asylums Board and Sick Children: Inhumanity or Progress?

*To the Editor of* The Hospital.

Sir,—Your comments on the recent circular of the Metropolitan Asylums Board advocating the admission of certain classes of children to the Carshalton and Park Hospitals and Dr. Morgan's criticism of those comments bring into prominence a matter of the very greatest importance to the poor of London. May I point out to begin with that Dr. Morgan does not seem to have read the paragraph he criticises very carefully? No general objection is taken in it to the removal of the cases to the Board's Hospitals for Poor-Law children—on the contrary, the suggestion is approved of in respect to four out of the five classes enumerated, but with regard to the fifth class, the proposal that children should be removed for operation to a hospital situated at a distance from their homes when such operation could be conducted equally well in the local Poor-Law infirmary is stigmatised as "rather inhuman."

Personally, I fully agree with the comments in your paragraph. Except for a few London Unions, the local Poor-Law infirmaries are as well and often better equipped for operative surgery than are the hospitals of the Metropolitan Asylums Board. Such being the case there is no advantage in sending a child a distance from its home when it can be operated upon under equally good conditions close by. On the contrary, it is a matter of the greatest importance both to the child and to the parents that during the anxious time preceding and following an operation the parents should be in a position to see their child frequently. In such a case, especially with parents to whom a tram fare is a consideration, the matter of distance is very far from being a puerile objection. Suggest to such a parent that his child, suffering, say, from appendicitis, should be taken to a hospital ten or fifteen miles off when it can be operated upon at a hospital within a mile or two of the home, and I doubt if he would consider "rather inhuman" a phrase strong enough to apply to such a proposal.

There remain those few infirmaries where proper provision for operative surgery has not been made. But here the remedy surely is to make the Guardians meet their responsibilities. The whole matter is another illustration of the need of a central authority which would co-ordinate the manifold activities at work in London for the medical relief of the sick under the Poor Law, and would ensure each institution carrying out the work for which it is best suited and placed.—Yours faithfully,

C. Thackray Parsons,
Medical Superintendent Fulham Infirmary.

